# Gray Matter Volume and Resting-State Functional Connectivity of the Motor Cortex-Cerebellum Network Reflect the Individual Variation in Masticatory Performance in Healthy Elderly People

**DOI:** 10.3389/fnagi.2015.00247

**Published:** 2016-01-07

**Authors:** Chia-Shu Lin, Shih-Yun Wu, Ching-Yi Wu, Hsien-Wei Ko

**Affiliations:** ^1^Department of Dentistry, School of Dentistry, National Yang-Ming University, Taipei, Taiwan; ^2^Division of Family Dentistry, Department of Stomatology, Taipei Veterans General Hospital, Taipei, Taiwan; ^3^Institute of Oral Biology, School of Dentistry, National Yang-Ming University, Taipei, Taiwan

**Keywords:** functional magnetic resonance imaging, masticatory performance, cerebellum, premotor cortex, voxel-based morphometry, resting-state functional connectivity

## Abstract

Neuroimaging studies have consistently identified brain activation in the motor area and the cerebellum during chewing. In this study, we further investigated the structural and functional brain signature associated with masticatory performance, which is a widely used index for evaluating overall masticatory function in the elderly. Twenty-five healthy elderly participants underwent oral examinations, masticatory performance tests, and behavioral assessments, including the Cognitive Abilities Screening Instrument and the short-form Geriatric Depression Scale. Masticatory performance was assessed with the validated colorimetric method, using color-changeable chewing gum. T1-weighted structural magnetic resonance imaging (MRI) and resting-state function MRI were performed. We analyzed alterations in gray matter volume (GMV) using voxel-based morphometry and resting-state functional connectivity (rsFC) between brain regions using the seed-based method. The structural and functional MRI analyses revealed the following findings: (1) the GMV change in the premotor cortex was positively correlated with masticatory performance. (2) The rsFC between the cerebellum and the premotor cortex was positively correlated with masticatory performance. (3) The GMV changes in the dorsolateral prefrontal cortex (DLPFC), as well as the rsFC between the cerebellum and the DLPFC, were positively correlated with masticatory performance. The findings showed that in the premotor cortex, a reduction of GMV and rsFC would reflect declined masticatory performance. The positive correlation between DLPFC connectivity and masticatory performance implies that masticatory ability is associated with cognitive function in the elderly. Our findings highlighted the role of the central nervous system in masticatory performance and increased our understanding of the structural and functional brain signature underlying individual variations in masticatory performance in the elderly.

## Introduction

Masticatory performance is a widely used objective measure of the clinical ability for food comminution (The Academy of Prosthodontics, 2005). It indexes the overall conditions of mastication, including the number of functional teeth, the maximal bite force, and the saliva flow rate (Ikebe et al., 2011). Declined masticatory performance has been associated with aging oral conditions (Hatch et al., 2001) and subjective dissatisfaction about masticatory function (Ikebe et al., 2007b). Therefore, assessing masticatory performance is critical to prosthodontic treatment of the elderly (Wayler and Chauncey, 1983). In addition to the peripherally oral conditions, mastication is a highly coordinated movement regulated by the central nervous system (Avivi-Arber et al., 2011). Neuroimaging studies have consistently revealed age-related alterations in intrinsic brain function and structure, including gray matter volume (GMV) (Smith et al., 2007) and resting-state functional connectivity (rsFC) (Ferreira and Busatto, 2013). Therefore, in the elderly, declined masticatory performance may be associated with changes in brain structure and/or function. Until now, the intrinsic brain signature of masticatory performance in the elderly has been unclear.

Neuroimaging studies have shown that chewing is predominantly associated with brain activity of the motor area and the cerebellum (Onozuka et al., 2002; Quintero et al., 2013a). The motor area is pivotal to initiate and represent motor program (Huber et al., 2012). The cerebellum plays a key role in controlling and learning movement (Tanji, 2001). It is important to note that the activity of both regions is age related (Langan et al., 2010; Boisgontier, 2015). Therefore, both the motor area and the cerebellum are candidate brain regions in which structural and functional changes would reflect individual variations in masticatory performance. Notably, evidence from animal models has shown that alterations in oral function (e.g., chewing and swallowing) conversely reshape the brain regions related to oral sensorimotor functions, a process known as brain neuroplasticity (Avivi-Arber et al., 2011; Zatorre et al., 2012). These findings imply that on the one hand, the intrinsic difference in brain signature may contribute to individual variations of masticatory function. On the other hand, long-term experience with mastication may conversely reshape the brain.

The current study aimed to investigate the structural and functional brain signature of masticatory performance in the healthy elderly population. Here, we hypothesized that the motor area and cerebellum, which are the core regions of the cortico-cerebellar network of motor control and mastication (Quintero et al., 2013b), would contribute to individual variations in masticatory performance. Using structural and functional magnetic resonance imaging (MRI), we investigated (1) the GMV of the brain and (2) rsFC between brain regions. Reduced GMV is associated with an age-related decline in motor function (Smith et al., 2007). Changes in rsFC would reflect variations in motor function (Langan et al., 2010). Therefore, we specifically hypothesized that among elderly participants, (1) the GMV in the motor area and the cerebellum are correlated with masticatory performance, and (2) the rsFC change between the motor area and the cerebellum is correlated with masticatory performance.

## Materials and Methods

### Study Group

Twenty-seven elderly participants, who were 55 years of age or older and able to communicate with the experimenters, were recruited. The following exclusion criteria were applied: (1) a history of major physical or psychiatric disorders, including epilepsy, major depression, schizophrenia, or neurovascular diseases, (2) a history of brain injury or brain surgery, and (3) the inability to undergo an MRI due to physical or psychological contraindications. The participants were recruited via advertisements at local community centers and at the Taipei Veterans General Hospital. The study was approved by the Institutional Review Board of the National Yang Ming University and the Taipei Veterans General Hospital, Taiwan. Written informed consent was provided by all participants before the start of the experiment. One participant was excluded due to a failure of imaging acquisition, and one participant was excluded due to severe deterioration in masticatory performance (see Results), and therefore, 25 participants were included (Table 1).

**Table 1:** **Demographic, behavioral, and clinical profiles of the study group**.

		*N*	Mean	SD	Max	Min
**Demographic**						
Gender	Female	17				
	Male	8				
Age^a^			64.2	6.3	74	55
Education	University/college	7				
	Professional school	3				
	High school	10				
	Elementary school	5				
**Behavioral**						
CASI^a^			95.3	4.7	100	84
GDS			1.6	1.8	6	0
**Clinical**						
Prosthesis type	No prosthesis	7				
	Removable denture	5				
	Fixed prosthesis	13				
Eichner Index	A	15				
	B	8				
	C	2				
MPI^a^			70.3	3.3	75.6	63.5

*CASI, the Cognitive Abilities Screening Instrument; GDS, the Geriatric Depression Scale-short form (GDS); MPI, Masticatory Performance Index; *N*, number of participants*.

*^a^Data conform to a normal distribution, based on Ryan Joiner test*.

### Behavioral Assessment

Before the MRI scan, we used the Cognitive Abilities Screening Instrument (CASI) (Teng et al., 1994) to assess the participants’ basic cognitive abilities, including memory, language, attention, and abstract thinking. Participants with impaired cognitive abilities would be further screened. We used the Geriatric Depression Scale-short form (GDS) (Sheikh and Yesavage, 1986) to screen out participants with severe depression, which could influence the intrinsic brain function and structure (Bora et al., 2012). All the assessments were performed in a quiet room at the 3T MRI laboratory. The GDS is a self-rated questionnaire. The CASI assessment was performed by experimenters Chia-Shu Lin and Hsien-Wei Ko. Both experimenters were trained in the assessment at Taipei Veterans General Hospital, Taiwan. All assessments were performed using the corresponding validated Chinese versions (Liu et al., 1997; Lin et al., 2012).

### Clinical Assessment

Here, we used the colorimetric method based on color-changeable chewing gum (Masticatory Performance Evaluating Gum XYLITOL, Lotte Co. Ltd., Tokyo, Japan) to assess the participants’ masticatory performance (Hayakawa et al., 1998; Hama et al., 2014). Participants were asked to chew the gum for 3 min in the same way that they ordinarily eat food. After 3 min of chewing, the participant was asked to expectorate the chewed gum on a transparent film, and the gum was flattened to a 1-mm thick wafer by compression. Each side of the wafer (i.e., the flattened gum) was scanned with an image resolution of 300 dots per inch, and the digitized images were stored in a jpeg format. The protocol of digitization was adopted from a previously published method (Schimmel et al., 2007). Next, we measured the L*, a*, and b* values on the digitized images, based on the CIELAB color system, using colorimetric software (Aquarelle Picker v1.0, Aquarelle Soft). The system quantifies color change with three chromaticity coordinates: L* (lightness), a* (green–red), and b* (yellow–blue). For example, the higher the value of the chromaticity coordinate a*, the higher the degree of red is. From the digitized image, we selected five points and assessed the L*, a*, and b* values of the points. These five points include one point at the center and the other four near the top, the bottom, the left, and the right margins (Hayakawa et al., 1998; Hama et al., 2014). Because two images were scanned for each side of the wafer, a total of 10 points were selected. The averages of the values from the 10 points were designated as the L*, a*, and b* values of the participant. Finally, the overall degree of color change (ΔE) was calculated by the following formula, according to Hama et al. (2014):

$$\Delta \text{E}=\sqrt{{\left({\text{L}}^{*}-80.1\right)}^{2}+{\left({\text{a}}^{*}+21.0\right)}^{2}+{\left({\text{b}}^{*}-40.2\right)}^{2}}$$

Here, 80.1 ± 0.7, −21.0 ± 1.3, and 40.2 ± 0.9 were mean ± SD of the L*, a*, and b* values, respectively, obtained from five pieces of the gum before chewing. The ΔE value, which denotes the color change from before to after chewing, is used as the masticatory performance index (MPI) (Hama et al., 2014).

Because the selection of regions is subjective, we performed two analyses to investigate the inter-rater reliability and the intra-rater test–retest reliability of the procedure. The analyses were performed using IBM SPSS Statistics 20. For the inter-rater reliability, two experimenters (Chia-Shu Lin and Hsien-Wei Ko) independently analyzed the MPI. Agreement of the two series of results was evaluated using the intra-class correlation coefficient (ICC) model, which assesses the absolute agreement of rating between two raters based on a two-factor mixed model. The analysis revealed high inter-rater reliability (averaged ICC = 0.978). For the test–retest reliability, one experimenter (Chia-Shu Lin) analyzed the MPI twice, with a break of 5 days between analyses. The test–retest reliability of the two series of results was evaluated using the ICC model, which assesses the consistency of rating between two time points based on a two-factor mixed model The analysis revealed high test–retest reliability (averaged ICC = 0.986).

The oral examinations were performed by a dentist (Chia-Shu Lin). For all participants, the Eichner Index (Eichner, 1955) was calculated based on the pattern of posterior contact of functional teeth, including natural and treated teeth, pontics in fixed prostheses, and implant-supported dental prostheses.

### Acquisition of Imaging Data

T1-weighted MRI and resting-state functional MRI data were acquired at the MRI Laboratory of National Yang-Ming University using a 3-Tesla Siemens MRI scanner (Siemens Magnetom Tim Trio, Erlangen, Germany). The total scan time was approximately 11.5 min. For all 25 participants, high-resolution T1 structural images were acquired in the sagittal plane using a high-resolution sequence [(TR) = 2530 ms, (TE) = 3.02 ms, matrix size = 256 × 256 × 192, voxel size = 1 mm × 1 mm × 1 mm]. Because of busy personal schedules, five participants only received the T1 scan. Twenty participants also underwent a resting-state functional MRI (fMRI) scan using a gradient echo EPI (Echo Planar Imaging) T2* weighted sequence [(TR) = 2000 ms, (TE) = 20 ms, matrix size = 64 × 64 × 40, voxel size = 3.4 mm × 3.4 mm × 3.4 mm, 183 volumes in total]. During scanning, the participants were instructed to relax, remain awake, and keep their eyes open and fixed on a cross symbol on the screen.

### Imaging Data Processing

#### T1-Weighted MRI

We applied voxel-based morphometry (VBM) to quantify GMV for individual participants, using the DARTEL (Diffeomorphic Anatomical Registration Through Exponentiated Lie Algebra) package (Ashburner, 2007) of SPM8 (Statistical Parametric Mapping, http://www.fil.ion.ucl.ac.uk/spm). The initial T1 images were segmented into gray matter (GM), white matter, and cerebrospinal fluid segments with a default 0.0001 bias regularization and warped to an ICBM (International Consortium for Brain Mapping) template for East Asian brains. A customized template was created using the GM segmented images from all 25 participants. Subsequently, the individual GM images were registered to the template and normalized to the Montreal Neurological Institute (MNI) space. Finally, they were spatially smoothed with an 8 mm full-width at half maximum (FWHM) Gaussian kernel. The resulting normalized and smoothed GM images were used for the VBM analyses.

#### Resting-State Functional MRI

Pre-processing of the resting-state fMRI data was performed using the Data Processing Assistant for Resting-State fMRI (Chao-Gan and Yu-Feng, 2010) and the Resting-State fMRI Data Analysis Toolkit (Song et al., 2011). The first three scans were discarded due to magnetic saturation effects. The imaging data were corrected for slice-timing order and head movement and were normalized to the MNI template. No participant showed excessive head movement (head motion of any volume >2.5 mm or 2.5°). The time series was band-pass filtered (0.01–0.08 Hz) to extract the low-frequency oscillating components of functional connectivity. The following factors were regressed out for spurious or non-specific effects: (a) the six movement parameters computed based on rigid body translation and rotation in pre-processing, (b) the mean signal within the lateral ventricles, (c) the mean signal within the deep white matter, and (d) the global mean signal. The processed imaging data were subsequently used for the seed-based functional connectivity (SBFC) analysis.

### Statistical Analysis

#### Voxel-Based Morphometry for Gray Matter Volume

The pre-processed GM images were analyzed using general linear models (GLMs) to assess the association between GMV and the MPI for each brain voxel. The MPI, age, gender, and total brain volume (TBV) were included as the covariates of the model. The last three covariates were considered as the nuisance factors that may influence individual GMV (Barnes et al., 2010), and therefore, their effects were removed in the model.

We first restricted the VBM analyses to the regions of interest (ROIs) in the motor area and cerebellum, with small volume correction. The ROIs were defined according to a previous study on the age-related brain mechanisms of mastication and aging (Onozuka et al., 2003) (see Tables 2 and 3 for the ROIs). The results were considered statistically significant if the *p* value corrected for family wise error (*P*_FWE_) <0.05. We also performed an exploratory whole-brain analysis with a lenient statistical threshold: uncorrected *p* value (P_unc_) <0.001 and cluster size >5 voxels. A suprathreshold brain region denotes that the regional GMV is significantly positively correlated with the MPI.

**Table 2:** **List of regions of interest based on Onozuka et al. (2003) Table 1, *the aged subjects***.

Regions of interest	MNI coordinates^a^		
	*x*	*y*	*z*
Sensorimotor cortex	2	−13	76
Supplementary motor area	62	−3	14
Cerebellum	24	−64	−30

*All ROIs were created as a sphere centered at the loci, with a radius = 8 mm*.

*^a^The Talairach coordinates that were originally reported were converted to MNI coordinates in this table based on the Brett transformation using *mni2tal.m* (http://imaging.mrc-cbu.cam.ac.uk/imaging/MniTalairach)*.

**Table 3:** **The brain regions where gray matter volume was positively correlated with the masticatory performance index**.

Brain region^a^	Side	Cluster size (voxels)	*Z* score	*P* value	MNI coordinates		
					*x*	*y*	*z*
Premotor cortex	R	81	4.0	<0.001	24	−7	66
Dorsolateral prefrontal cortex	L	37	3.9	<0.001	−24	21	51
Cuneus	R	36	3.7	<0.001	8	−75	23
Insula	L	27	3.7	<0.001	−45	−1	9
Inferior temporal gyrus	R	41	3.6	<0.001	35	−1	−44
Precentral gyrus	R	27	3.3	<0.001	47	0	48

*^a^The labels of brain regions were surveyed using the Harvard-Oxford cortical and subcortical structural atlases and the Jülich histological atlas of FSLView v3.1 (http://fsl.fmrib.ox.ac.uk/fsl/fslview)*.

#### Seed-Based Functional Connectivity Analysis

The VBM analyses revealed that GMV of the premotor cortex and the precentral gyrus was positively correlated with the MPI (Tables 2 and 3). Subsequently, we investigated the rsFC of these regions as the seed ROIs. To test our second hypothesis, for each seed ROI, a GLM was modeled to investigate the correlation between the rsFC of the seed ROI and the MPI. Age and gender were modeled as nuisance covariates. For exploratory purposes, we also applied a lenient statistical threshold: P_unc_ <0.001 and cluster size >5 voxels. A suprathreshold brain region indicates that its rsFC with the seed ROI is significantly positively correlated with the MPI.

Additionally, we found that GMV of the dorsolateral prefrontal cortex (DLPFC) was positively correlated with the MPI. Due to its role in cognitive processing and motor control (Rowe et al., 2006), we performed an *ad hoc* SBFC analysis, using the DLPFC as a seed, with the same statistical threshold.

## Results

### Behavioral and Clinical Assessments

The demographic, behavioral, and clinical profiles of the study group are shown in Table 1. Behaviorally, all participants showed normal cognitive abilities (CASI >80) and no signs of depression (GDS <7) based on the local normative data (Chang and Tsai, 2003; Lin et al., 2012). Clinically, most of the participants (60%) were categorized as Class A by the Eichner index (*n* = 15), indicating adequate posterior contact. Five participants were categorized as Class B3/B4/C, all of whom wore a removable denture with sufficient posterior contact. One participant with a very low MPI (53.1), due to severe tooth loss and lack of a denture, was excluded as an outlier (i.e., an MPI lower than mean-2 × SD MPI). Further analyses of the Pearson correlation coefficient revealed that age was significantly negatively correlated with the MPI (*r* = −0.54, two-tailed *p* = 0.005), which was consistent with previous findings (Ikebe et al., 2011).

### The VBM Analyses

**Figure 1:**
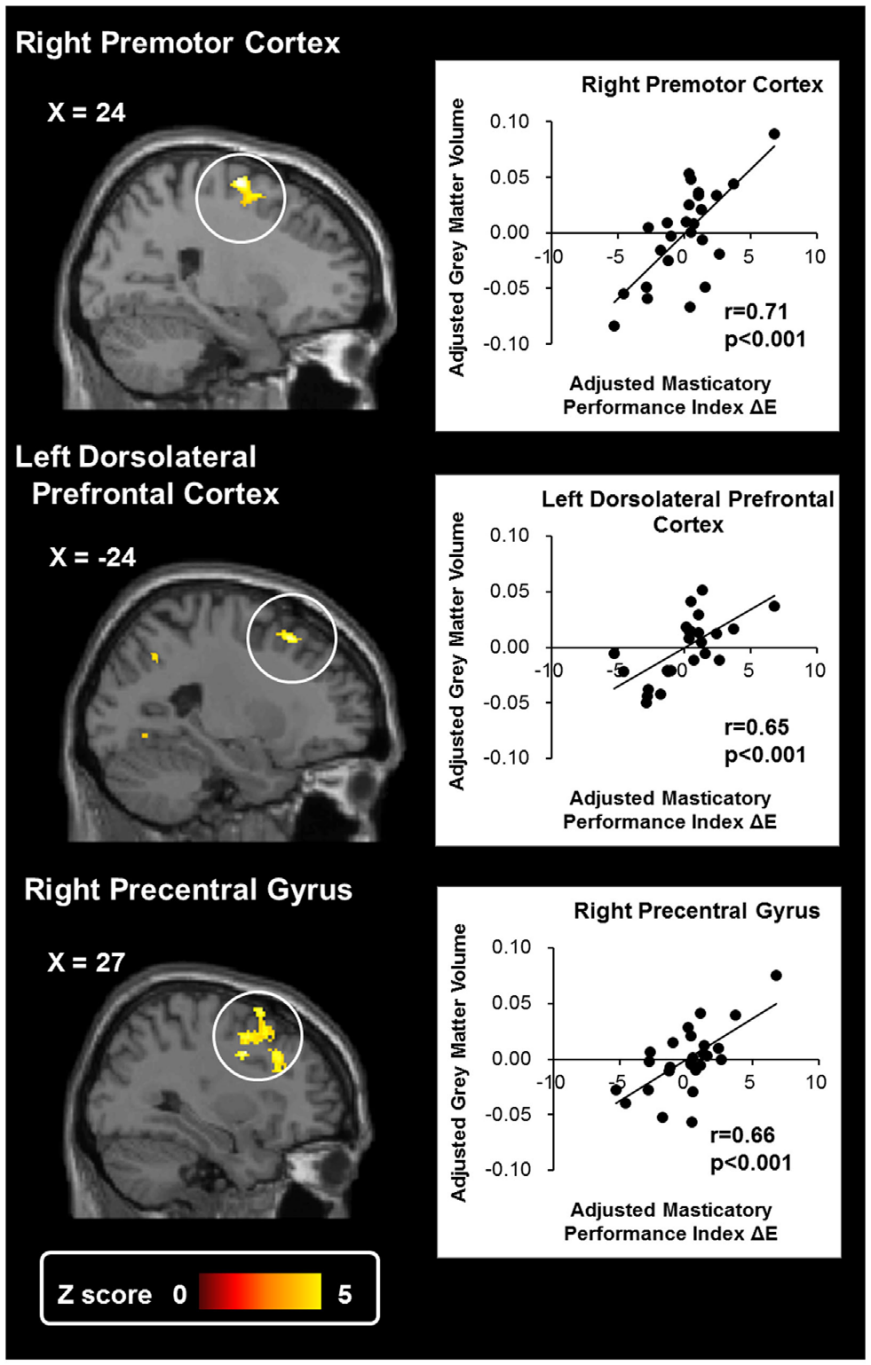
**Results of the voxel-based morphometry of gray matter volume**. The whole-brain exploratory analysis revealed significant activation at the right premotor cortex, the left dorsolateral prefrontal cortex, and the right precentral gyrus. The gray matter density of these regions was significantly positively correlated with masticatory performance. The scatter plots show the association between the masticatory performance index (ΔE) and the gray matter volume of each region. The masticatory performance index and gray matter volume were adjusted to remove the effects of age, gender, and total brain volume.

Tables 2 and 3 show the suprathreshold brain regions in the VBM analyses, i.e., the regions where the GMV was significantly positively correlated with the MPI. In terms of the ROI-based analysis, no suprathreshold region was found in the cerebellum or the motor area. The whole-brain exploratory analysis revealed suprathreshold regions in the right premotor cortex, the right precentral gyrus, the left DLPFC, the right cuneus, the left insula, and the right inferior temporal gyrus (Tables 2 and 3; Figure 1). These findings suggest that the GMV change in the motor area would reflect individual variations in masticatory performance.

### The SBFC Analyses

**Figure 2:**
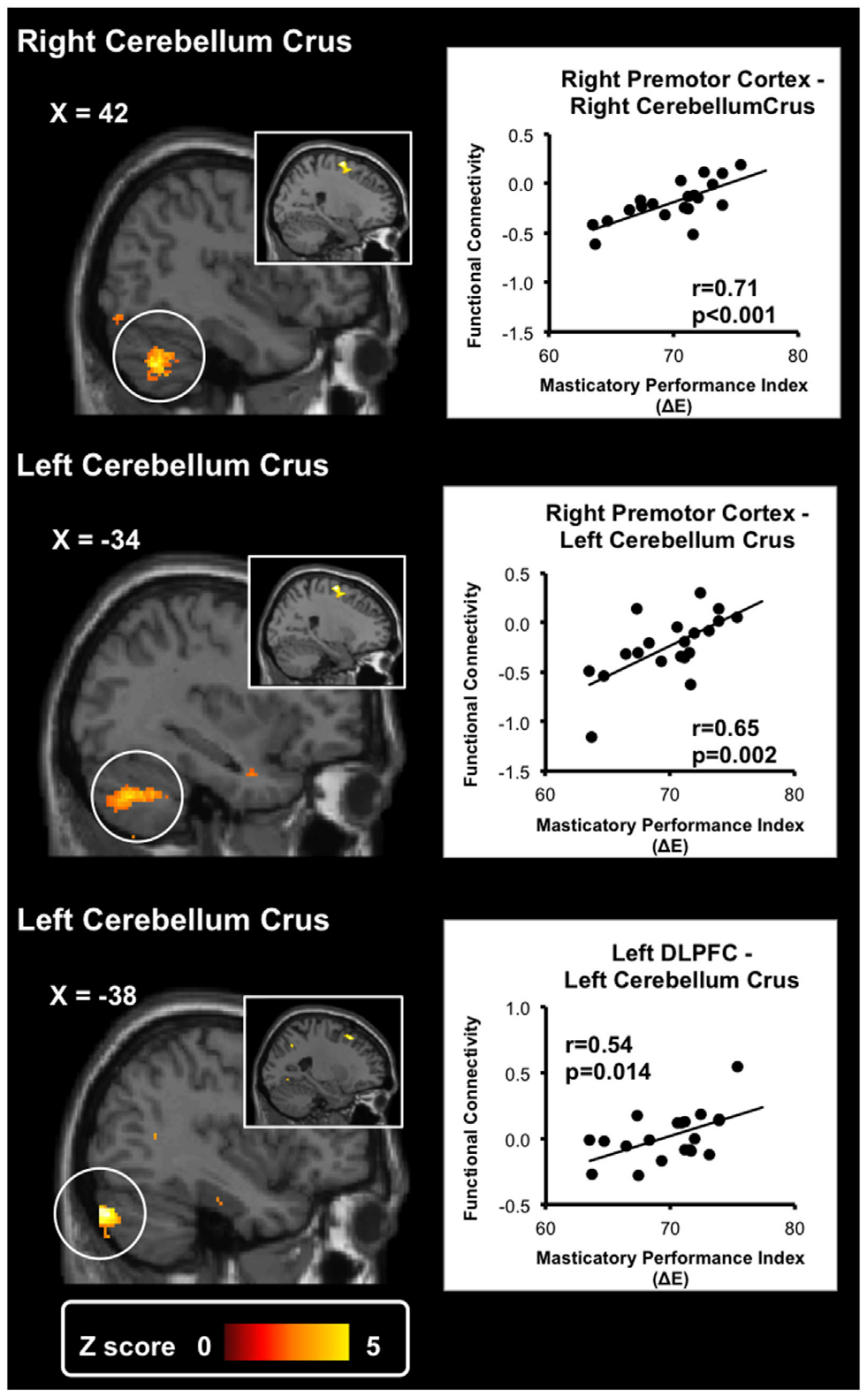
**Results of the seed-based functional connectivity analyses**. Two seed regions, the right premotor cortex and the left dorsolateral prefrontal cortex (shown in the inlet pictures), were defined based on the results of the voxel-based morphometry. The whole-brain exploratory analysis revealed significant activation in the bilateral cerebellum crus. Resting-state functional connectivity between the seed regions and the cerebellum crus was significantly positively correlated with masticatory performance. The scatter plots show the association between the masticatory performance index (ΔE) and the functional connectivity of each seed region. The functional connectivity was adjusted to remove the effects of age and gender.

Table 4 shows the results of the SBFC analyses. When the right premotor cortex was used as the seed ROI, we found suprathreshold regions in the bilateral cerebellum crus. The findings showed that the rsFC between the premotor cortex and the cerebellum was positively correlated with the MPI. When the right precentral gyrus was used as the seed ROI, we found suprathreshold regions in the dispersed cortical regions, including the temporal lobe, the orbitofrontal cortex, and the fusiform gyrus. Additionally, when the DLPFC was used as the seed ROI, we found a suprathreshold region in the bilateral cerebellum (Table 4; Figure 2). These findings suggest that the cerebellum-motor area connectivity would reflect individual variations in masticatory performance.

**Table 4:** **The brain regions where functional connectivity of the seed regions was positively correlated with the masticatory performance index**.

Brain region^a^	Side	Cluster size (voxels)	*Z* score	*P* value	MNI coordinates		
					*x*	*y*	*z*
**Seed region: right premotor cortex**							
Middle temporal gyrus	R	93	4.6	<0.001	64	−30	−14
Cerebellum crus I	R	116	4.0	<0.001	42	−60	−42
Superior parietal lobe	L	23	3.6	<0.001	−12	−68	64
Lateral occipital lobe	R	11	3.5	<0.001	36	−82	−14
Cerebellum crus I	L	36	3.5	<0.001	−34	−64	−38
Cerebellum lobule VIIb	L	8	3.4	<0.001	−42	−60	−56
Cerebellum lobule VIIb	R	5	3.4	<0.001	16	−74	−54
**Seed region: dorsolateral prefrontal cortex**							
Cerebellum crus I	L	81	4.4	<0.001	−38	−82	−32
Cerebellum crus II	L	29	3.9	<0.001	−46	−78	−44
Cerebellum lobule VI	L	43	3.9	<0.001	−22	−60	−16
Frontal pole	R	22	3.8	<0.001	12	50	50
Cerebellum lobule VIIbs	R	7	3.7	<0.001	10	−80	−52
**Seed region: right precentral gyrus**							
Inferior temporal gyrus	L	14	3.5	<0.001	−42	−10	−36
Orbitofrontal cortex	L	11	3.5	<0.001	−14	6	−22
Fusiform gyrus	L	17	3.4	<0.001	−22	−78	−16
Visual cortex	L	5	3.4	<0.001	−46	−80	4
Temporal pole	L	5	3.3	<0.001	−26	12	−36

*^a^The labels of brain regions were surveyed using the Harvard-Oxford cortical and subcortical structural atlases and the Jülich histological atlas of FSLView v3.1 (http://fsl.fmrib.ox.ac.uk/fsl/fslview)*.

## Discussion

Previous studies have identified brain activation in the cerebellum and the motor cortex when participants are chewing (Onozuka et al., 2002; Quintero et al., 2013a). The studies have shown the activity-evoked pattern of brain activity. Our study extends this scope by providing novel evidence on the *intrinsic* brain signatures, i.e., the individual structural and functional difference, underlying the individual variation in masticatory performance, which is a critical index for evaluating oral function in the elderly. Our structural and functional MRI analyses revealed the following findings:

1. Change in GMV in the premotor cortex and the precentral gyrus is positively correlated with masticatory performance (Tables 2 and 3; Figure 1).
2. Change in rsFC between the cerebellum and the premotor cortex is positively correlated with masticatory performance (Table 4; Figure 2). The findings showed that in the premotor cortex, reductions of GMV and rsFC would reflect a decline in masticatory performance.
3. Gray matter volume changes in the DLPFC, as well as the rsFC between the cerebellum and the DLPFC, were positively correlated with masticatory performance (Table 4; Figure 2). The findings echoed the hypothetical link between mastication and cognitive function in the elderly (Weijenberg et al., 2011).

The VBM analyses revealed that GMV of the premotor cortex and precentral gyrus (close to the premotor cortex) is positively associated with masticatory performance (Tables 2 and 3; Figure 1). The premotor cortex predominantly subserves the selection and planning of movement and plays a key role in bridging information between the primary motor cortex (M1) and the prefrontal cortex (Wise, 1985). Notably, premotor activation is modulated by increased attention to action (Rowe et al., 2002). During movement, the elderly recruit more extensive premotor cortex activity compared to younger people, suggesting a greater effort in motor control (Rowe et al., 2006). Furthermore, the premotor cortex is critical to polymodal motion processing, i.e., an integration of visual, tactile, vestibular sensations with movement (Bremmer et al., 2001). Polymodal integration is critical to mastication, which demands jaw movement and oral tactile stereognosis (Ikebe et al., 2007a). Therefore, reduced premotor GMV may reflect reduced abilities in motor, attention, and sensory functions, which are associated with poorer masticatory performance.

In contrast, we did not find significant results in the M1. The M1 initiates and maintains voluntary movement, and therefore, its activation has been observed during chewing (Onozuka et al., 2002; Quintero et al., 2013a). The current study, in contrast, investigated the brain signature during a resting state, and therefore, we did not identify significant M1 activation. Our findings suggest that in terms of masticatory performance, planning, and control of movement (as directed by the premotor cortex) would play a more significant role than initiation and retention of movement (as directed by the M1).

The SBFC analyses revealed that the connectivity between the cerebellum and the premotor cortex were positively correlated with masticatory performance (Table 4; Figure 2). Consistently, when an individual was chewing, there was increased functional connectivity between the cerebellum and the motor area (Quintero et al., 2013b). The cerebellum plays a key role in coordinating and monitoring rhythmic movements, such as walking and chewing (Galea et al., 2011). It receives input from the brain stem central pattern generator and connects with the sensorimotor cortex, playing a pivotal role the neural circuitry of mastication (Avivi-Arber et al., 2011). Therefore, the reduced premotor cortex-cerebellum rsFC may indicate less coupled coordination of motor control, which leads to impaired motor control and a decline in masticatory performance.

While we found that the cerebellum-motor rsFC was correlated with masticatory performance, the cerebellar GMV *per se* was not. The aging-related death of cerebellar neurons leads to impaired motor control (Boisgontier, 2015), such as masticatory dyspraxia (Marien et al., 2013). Therefore, changes in cerebellar GMV may be associated with more extensive functional impairment, which was not expected in the physically healthy, elderly participants recruited in this study.

The relationship between mastication and cognitive change has been a topic of much debate [for a review see (Weijenberg et al., 2011)]. In aged mice, the molarless condition or long-term soft-diet feeding which reduced chewing ability would attenuate spatial learning/navigation (Watanabe et al., 2002; Tsutsui et al., 2007) and the ability of object discrimination (Kawahata et al., 2014). In rats, tooth loss would contribute to a decrease in the hippocampal cell density and impaired spatial learning (Yamazaki et al., 2008). The evidence from animal studies has revealed that an intact masticatory ability may play a key role in cognition. We found that masticatory performance was correlated with changes in connectivity between the cerebellar crus and the DLPFC (Table 4; Figure 2). In the elderly, a decreased cerebellar GMV is associated with physical frailty, including slow walking speed, muscle weakness, and low physical activity (Chen et al., 2015). The bilateral cerebellum crus is also associated with non-motor input, and its activation is associated with cognitive functions (Stoodley et al., 2012). It should be noted that the mastication-cognition association can be confounded by a variety of factors, such as daily living activities, nutritional status, and oral health conditions (Weijenberg et al., 2011). Still, our findings suggest that individual variations in masticatory function are associated with the brain’s connectional architecture. Two related hypotheses remained to be tested: (1) on the one hand, the DLPFC may contribute to additional motor control on chewing activity, in order to compensate the age-related decrease in cerebellar motor function (Chen et al., 2015). (2) On the other hand, our finding may suggest that a decreased masticatory ability is associated with cognitive dysfunction (Ono et al., 2010) as predicted by the animal models (Watanabe et al., 2002; Tsutsui et al., 2007; Yamazaki et al., 2008; Kawahata et al., 2014). Further investigation is required to clarify this cause-effect relationship.

To our knowledge, this is the first study to investigate the structural and functional brain signature related to masticatory performance in the elderly. Due to the sampling and experimental methods, our findings should be considered with the following limitations:

1. We focused on elderly individuals who were physically and mentally healthy. Therefore, the correlation between the GMV change and masticatory performance is limited to healthy aging. The interplay between aging and pathological factors related to motor control (e.g., frailty) remains unclear.
2. We used masticatory performance as a clinical index for the overall ability to masticate. It should be noted that masticatory performance can be influenced by many factors, including saliva flow rate, occlusal force, and oral stereognosis (Ikebe et al., 2007a, 2011). More studies are required to clarify the association between the brain signature and the specific mastication-related factors.
3. The current study only performed correlation analyses for investigating different brain regions’ GMVs and connectivity associated with masticatory performance. The statistically significant correlation cannot be inferred to be a cause-effect link. For example, it is uncertain whether the reduced cerebellar GMV leads to reduced masticatory function or vice versa. A longitudinal study that evaluates an interventional effect (e.g., denture wearing) on masticatory performance would be helpful to clarify the cause-effect link.

Clinically, our findings suggest that in addition to oral conditions (e.g., the number of functional teeth), age-related changes in the central nervous system also contribute to masticatory performance. Therefore, regarding prosthodontic treatment, special care is needed for elderly patients who show signs of cortico-cerebellar deficits. Clinically, our findings can be explained in two ways. On the one hand, because the premotor cortex is critical to motor control, a greater degree of premotor GMV suggests that the individual has a greater capability to control mastication. On the other hand, from the perspective of brain plasticity, the variation in GMV may be associated with the degrees of neurogenesis and synaptogenesis, which are reshaped by long-term skill learning (Zatorre et al., 2012). Therefore, the correlation shown here may also suggest an experience-dependent plasticity on cortical neurons, as sculpted by mastication. Overall, the neuroimaging findings revealed the structural and functional brain signature underlying individual variations in masticatory performance in the elderly, which highlights the role of the central nervous system in mastication.

## Author Contributions

C-SL, S-YW, and C-YW conceived and designed the research. C-SL (predominantly) and H-WK executed the experiment. C-SL (predominantly) and H-WK analyzed the data. C-SL (predominantly), S-YW, and C-YW wrote the paper. C-SL, S-YW, C-YW, and H-WK finalized the paper.

## Conflict of Interest Statement

The authors declare that the research was conducted in the absence of any commercial or financial relationships that could be construed as a potential conflict of interest.
